# Generation of a serum free CHO DG44 cell line stably producing a broadly protective anti-influenza virus monoclonal antibody

**DOI:** 10.1371/journal.pone.0183315

**Published:** 2017-09-14

**Authors:** Veronika Chromikova, Maria A. Zaragoza, Florian Krammer

**Affiliations:** 1 Department of Microbiology, Icahn School of Medicine at Mount Sinai, New York, New York, United States of America; 2 Graduate School of Biomedical Sciences, Icahn School of Medicine at Mount Sinai, New York, New York, United States of America; Institute of Molecular and Cell Biology, SINGAPORE

## Abstract

Because of the broad neutralization and *in vivo* protection across influenza A and influenza B virus strains, monoclonal antibody CR9114 is widely used in influenza virus research as a positive control in many experiments. To produce amounts sufficient for the demand requires regular transient transfections, resulting in varying yield as well as differing batch to batch quality. Here, we report the development of a serum-free CHO DG44 cell line, stably producing a CR9114-like antibody with a potential to become a useful influenza virus research tool.

## Introduction

Monoclonal antibody (mAb) CR9114 was first reported by Dreyfus *et al*. in 2012 in a study describing broadly neutralizing human antibodies against influenza B virus strains [1]. What made this antibody so unique is its ability to bind to group 1 (H1, H2, H5, H6, H8, H9, H11, H12, H13, H16, HL17, HL18) and group 2 (H3, H4, H7, H10, H14, H15) influenza A virus hemagglutinins (HA) in addition to influenza B HA by interacting with a highly conserved epitope in the stalk region of HA. MAb CR9114 is using the V_H_1-69 germline gene and compared to other broadly neutralizing antibodies has a relatively small number of somatic mutations [1]. Remarkably, *in vitro* neutralization can only be observed with influenza A viruses while the mAb protects *in vivo* across a panel of both influenza A and influenza B strains [1]. This *in vivo* protection in the absence of neutralization is likely due to Fc-mediated functions and possibly neuraminidase inhibition [2]. For the above mentioned reasons this antibody is in high demand in the influenza virus research community and many laboratories use mAb CR9114 as a positive control in their experiments [3, 4], as a competition antibody in enzyme linked immunosorbent assays (ELISA) [5] or as a comparison when characterizing new broadly neutralizing antibodies [6]. Production of mAb by recombinant expression using transient transfections of cells can lead to a batch to batch variability and the mAb yield is influenced by the passage number and quality of the cultured cells, transfection efficiency and the toxicity of transfection reagents. Additionally, many (commonly available) cell lines used for transient transfection are serum-dependent, resulting in contamination of produced mAbs by bovine IgGs. Here, we decided to take advantage of a biotechnology-relevant production cell line, Chinese hamster ovary (CHO) DG44, to establish a serum-free, stable, CR9114-like (CR9114L, a generic version of CR9114) antibody-producing cell line for a steady supply of this mAb with low batch to batch variation. The CHO DG44 cell line, in which both alleles are dihydrofolate reductase (DHFR) negative, was created in 1980ies by ionizing radiation [7]. DHFR catalases reduction of dihydrofolic acid to tetrahydrofolic acid, which is required in the process of *de novo* synthesis of purines, thymidilic acid and certain amino acids. Therefore, CHO DG44 host cells medium requires supplementation by hypoxanthine and thymidine (HT). In recombinant cell lines, exogenous *dhfr* (together with the gene of interest, (GOI)) is provided by a vector used for transfection, making additional supplementation by HT unnecessary. Methotrexate (MTX) is an inhibitor of DHFR, often used to provide an amplification pressure to force cells into increasing the copy numbers of *dhfr* and GOI. Nowadays, CHO DG44 and CHO DUXB11 are two of the most widely used CHO cell lines, and CHO cells, in general, have become a work-horse of biopharmaceutical production, delivering 3-10g product per liter culture in highly optimized processes and surpassing some of the microbial systems [8]. A cell line that continuously expresses mAb CR9114L might be a very helpful tool for the academic influenza virus research community.

## Methods

### Cell culture and adaptation to serum-free conditions

CHO DG44 cells were obtained as a gift from Dr. Yiannis Ioannou at the Department of Genetics and Genomic Sciences at the Icahn School of Medicine at Mount Sinai and were routinely cultured in Dulbecco’s Modified Eagle’s Medium (DMEM)/Ham’s F12 medium (Life Technologies/Gibco) supplemented with 10% fetal bovine serum (FBS, HyClone), 2mM L-glutamine, 1x HT (hypoxanthine and thymidine) Supplement and Pen-Strep (penicillin [100 U/mL] and streptomycin [100μg/mL], Gibco). Sequential adaptation to serum-free medium started by decreasing the FBS content to 5%, and then subsequently to 2.5% and 2%. At 2% FBS concentration and below, the medium was additionally supplemented with 1x ITS (insulin, transferrin, and selenite) supplement, 1x polyamine supplement, 1x antioxidant supplement (all from Sigma) and 0.072 μg/mL hydrocortisone (Sigma). To proceed from 2% to 1% FBS content (and ultimately to serum-free conditions) CHO-S-SFM II (Gibco), CD OptiCHO (Gibco), ProCHO5 (Lonza) and CD DG44 (Gibco) media were tested, all supplemented as described above. Specific growth rate was calculated as $\frac{\text{ln}\left(max.\;cell\;density\right)-\text{ln}\left(cell\;density\;at\;seeding\right)}{\#\;days\;from\;last\;passage}$.

Expi293F cells were routinely cultivated in the Expi293F media (Gibco) at 37°C, 150 rpm in 7.5% CO_2_ incubator, using 100 mL shake flasks (Corning) and split twice a week. Madin-Darby canine kidney (MDCK) cells were routinely cultivated in DMEM supplemented with 10% FBS and Pen-Strep.

### Plasmid preparation

The DHFR sequence (NCBI accession number NM010049.3) was synthesized and flanked with *Xba*I and *Not*I restriction sites. The obtained polymerase chain reaction (PCR) fragment (primer sequences available upon request) was cloned into the multiple cloning site (MCS) B of the pIRES vector using an In-fusion cloning kit (Clontech). The mAb CR9114 heavy chain (HC) and light chain (LC) genes were PCR amplified from IgG1-AbVec and Igλ-AbVec vectors respectively [9, 10] and flanked with *Nhe*I and *EcoR*I restriction sites. The CR9114 HC gene was then cloned into MCS A on a plasmid already containing the DHFR sequence to enable gene-amplification under the methotrexate (MTX) pressure. The LC gene was inserted into the MCS A of an empty pIRES vector. The AbVec vectors containing the heavy and light chain sequences of CR9114 mAb were kindly provided by the laboratory of Dr. Patrick Wilson (University of Chicago) and used for transient transfections of Expi293F cells using ExpiFectamin^™^ transfection kit (Gibco) according to the manufacturer’s instructions.

### Transfection and selection process for serum free CHO DG44

Cells were seeded into a 6-well plate at a density of 1x10^6^ cells/well twenty-four hours before transfection. Transfection was performed using Lipofectamine^®^ 2000 (Thermo Fisher Scientific) according to the manufacturer’s instructions and equal amounts of HC and LC plasmid were co-transfected. 48 hours post-transfection, cells were transferred into 96-well plates at 7x10^3^ cells/well in a selection medium (CHO-S-SFM II supplemented as described above, with additional G418 and 100 nM MTX, and without an HT supplement to start the selection process). Approximately 3 weeks post-transfection a qualitative ELISA was performed and based on the result, 84 selected clones were moved to 24-well plates for expansion and the MTX pressure was increased to 200 nM. A final clone was selected after additional rounds of cell culture expansion and ELISA testing. Cells were then grown at 37°C, 7.5% CO_2_ and 150 rpm in a shaking incubator. The selection medium was additionally supplemented with 1% Pluronic F68 to protect cells from mechanical stress. Specific growth rates were calculated as described above and the specific productivity (qp) was calculated as described in [11] and below:

$$qp=\;\frac{\Delta P}{CCD}1000000\;\left[pg.cells^{-1}days^{-1}\right],$$

where ΔP represents the accumulation of the product and CCD represents the cumulative cell days.

### ELISA

ELISA plates (Immulon^®^ 4HBX, Thermo Scientific) were coated with 2 μg/mL protein (goat anti-human lambda chain antibody or recombinantly expressed HA glycoprotein. Recombinant HAs included were derived from A/PR/8/34 (H1N1), A/California/04/09 (H1N1), A/Indiana/10/11 (H3N2), A/chicken/Netherlands/14015531/14 (H5N8) and A/chicken/Italy/13474/99 (H7N1) [12, 13]) and incubated at 4°C overnight. Next day, plates were incubated with 3% milk in phosphate-buffered saline (PBS, pH7.4)) with 0.1% Tween 20 (PBST) for 2 hours at room temperature (RT), shaking. After the plates were washed, cell culture supernatant was applied and incubated for 1 hour at RT, shaking. Goat anti-human Fab antibody conjugated to horse-radish peroxidase (Sigma) was used as a secondary antibody in 1:3000 dilution. O-phenylenediamine dihydrochloride (OPD) (Sigma) was provided as a substrate for visualization and plates were measured using a Synergy plate reader at an optical density of 490 nm.

### Protein purification

Cell culture supernatant from stable cell line was collected, filtered through a 0.22 um filter and purified on an AKTA pure L25 system through a HiTrap protein-G column (GE Healthcare). Protein was eluted from the column using 0.1 M glycine pH 2.7 and fractions were neutralized using 2M TrisHCl pH 10. They were pooled and the buffer exchange to PBS was performed using Amicon^®^ Ultra 15 Centrifugal filters (Millipore) with a 30K cut off.

### Microneutralization assay

MDCK cells were seeded into 96-well plates (Sigma) at 1.5x10^4^/well twenty-four hours prior to the experiment and cultivated at 37°C in 5% CO_2_ in a cell culture incubator overnight. Next day, two-fold serial dilutions of CR9114L mAb expressed by the stable cell line and CR9114 mAb expressed transiently in Expi293F cells were prepared in 96-well U-bottom well plate (Costar), starting at 10 μg/mL and incubated with 200 plaque forming units (PFU)/well of A/Vietnam/1203/04; PR8 6:2 (H5N1, multibasic cleavage site removed, low pathogenic virus in A/PR/8/34 backbone [14]) influenza virus, A/Netherlands/602/2009 (H1N1) or A/Philippines/2/1982 (H3N2—X-79) influenza virus at RT for 1 hour, shaking. Viruses for this assay were grown in 8–10 day old embryonated chicken eggs and titered using a plaque assay on MDCK cells. Cells were washed 1x with PBS (Life Technologies), 100 uL of an antibody-virus mixture was transferred into each well and the plate was incubated at 37°C, 5% CO_2_ for an hour. After the incubation time, cells were washed again 1x with PBS, antibody serial dilutions were added and incubated for 48 hours at 37°C, 5% CO_2_. The HA readout was used to compare the neutralization potential between the two antibodies. Briefly, 50uL/well of cell supernatants were moved to the V-shaped 96-well plate (Thermo Fisher Scientific) and 50 uL/well of 0.5% chicken red blood cells (RBC) in PBS were added. Plates were incubated for one hour at 4°C and the minimum neutralizing concentration was determined by the identification of the last well where hemagglutination did not occur.

### Antibody-dependent cell-mediated cytotoxicity (ADCC) reporter assay

The ADCC reporter assay was performed using an ADCC Reporter Bioassay Kit (Promega), according to the manufacturer’s instructions. Briefly, MDCK cells were plated at 30 000 cells/well into flat-bottom white 96-well plates. Twenty-four hours later, cells were infected with A/Vietnam/1203/04; PR8 6:2 (H5N1) influenza virus at a multiplicity of infection (MOI) of 5 and 24 hours post-infection, the medium was replaced by antibody dilutions in the ADCC assay buffer. Effector cells were added and incubated for 6 hours before the addition of Bio-Glo Luc Assay reagent and substrate. Luminescence was measured at Synergy plate reader. ADCC induction was calculated according to the manufacturer’s instructions as a fold induction over the control.

## Results

### Adaptation of CHO DG44 cell line to serum-free conditions

**Fig 1:**
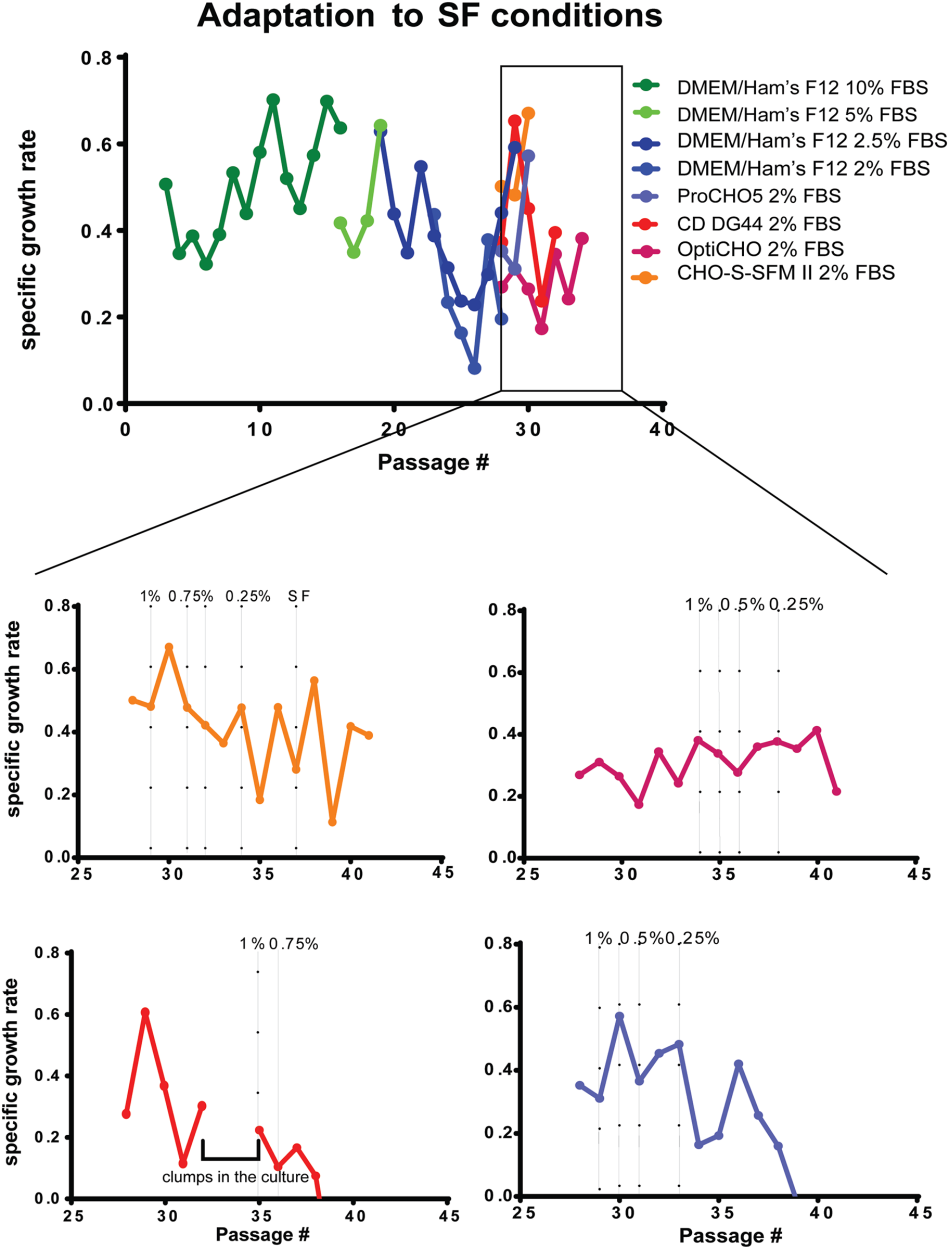
Overview of the specific growth rates during the individual steps of adaptation to serum-free (SF) conditions. Dark green curve represents standard cultivation conditions for the CHO DG44 cell line (DMEM/Ham’s F12 +10% FBS), other colors represent individual FBS-reduction steps according to the figure legend. At 2% FBS content, DMEM/Ham’s F12 media did not satisfy the needs of the culture despite added supplements. An additional set of commercially available media designed for CHO cultures was implemented for further FBS reduction steps as indicated (displayed in orange, magenta, red and purple in zoomed images).

CHO DG44 cells were cultivated in DMEM/Ham’s F12 medium supplemented with 10% FBS for 15 passages before the start of the adaptation to serum-free conditions. The viability of the cell culture was consistently above 90%. In a first step, the FBS content was reduced to 5%, which had a transient negative impact on the specific growth rate (Fig 1). The viability remained unchanged. Four passages later, the FBS content was further reduced to 2.5% which again slowed down the growth of the cells but eventually, they recovered again within the next 10 passages. As the FBS concentration was lowered to 2%, the DMEM/Ham’s F12 medium was supplemented as described above and in parallel ProCHO5, CD DG44, OptiCHO, and CHO-S-SFM II media (all of them supplemented the same way as DMEM/Ham’s F12) were tested (Fig 1). In each of these media, the FBS weaning process went on with FBS concentration lowered to 1%, 0.75%, 0.5%, 0.25% and finally FBS was removed entirely. The specific growth rate and cell viability were observed and CHO-S-SFM II medium was determined as the best option for cultivating this newly adapted CHO DG44 serum free (SF) cell line (Fig 1).

### Construction of expression vectors and establishing a stable cell line

**Fig 2:**
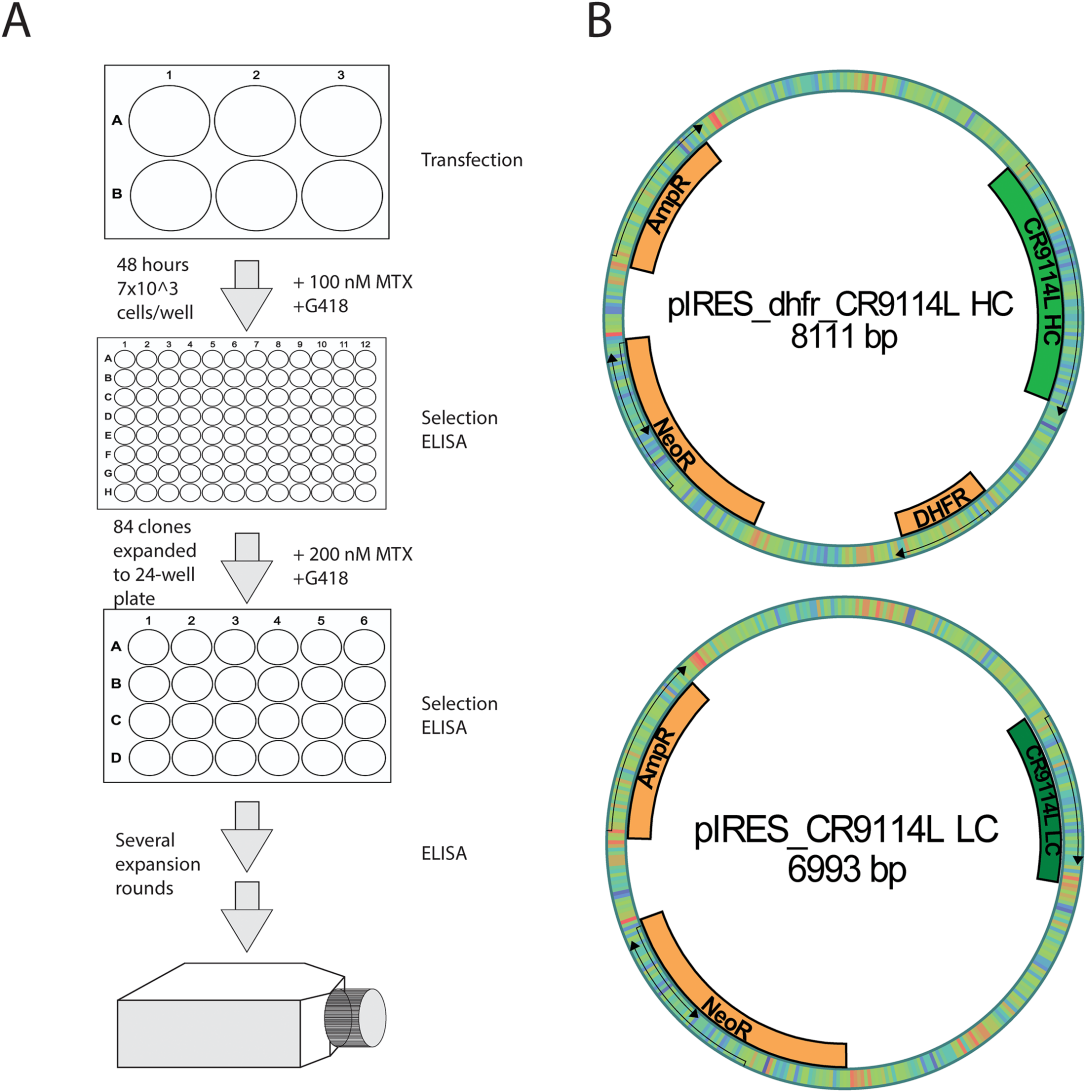
Visual overview of the selection and screening process and pIRES vector maps. A) Overview of the screening and selection process during the development of the stable recombinant 15G6 cell line. B) Heavy chain and light chain sequences of CR9114L mAb were cloned into pIRES vectors. HC-containing vector also contains a *dhfr* sequence enabling gene amplification in the presence of MTX. Color spectrum on the vector maps indicates GC content within the sequence (blue indicates high GC content). Vector maps were drawn in pDRAW32.

**Fig 3:**
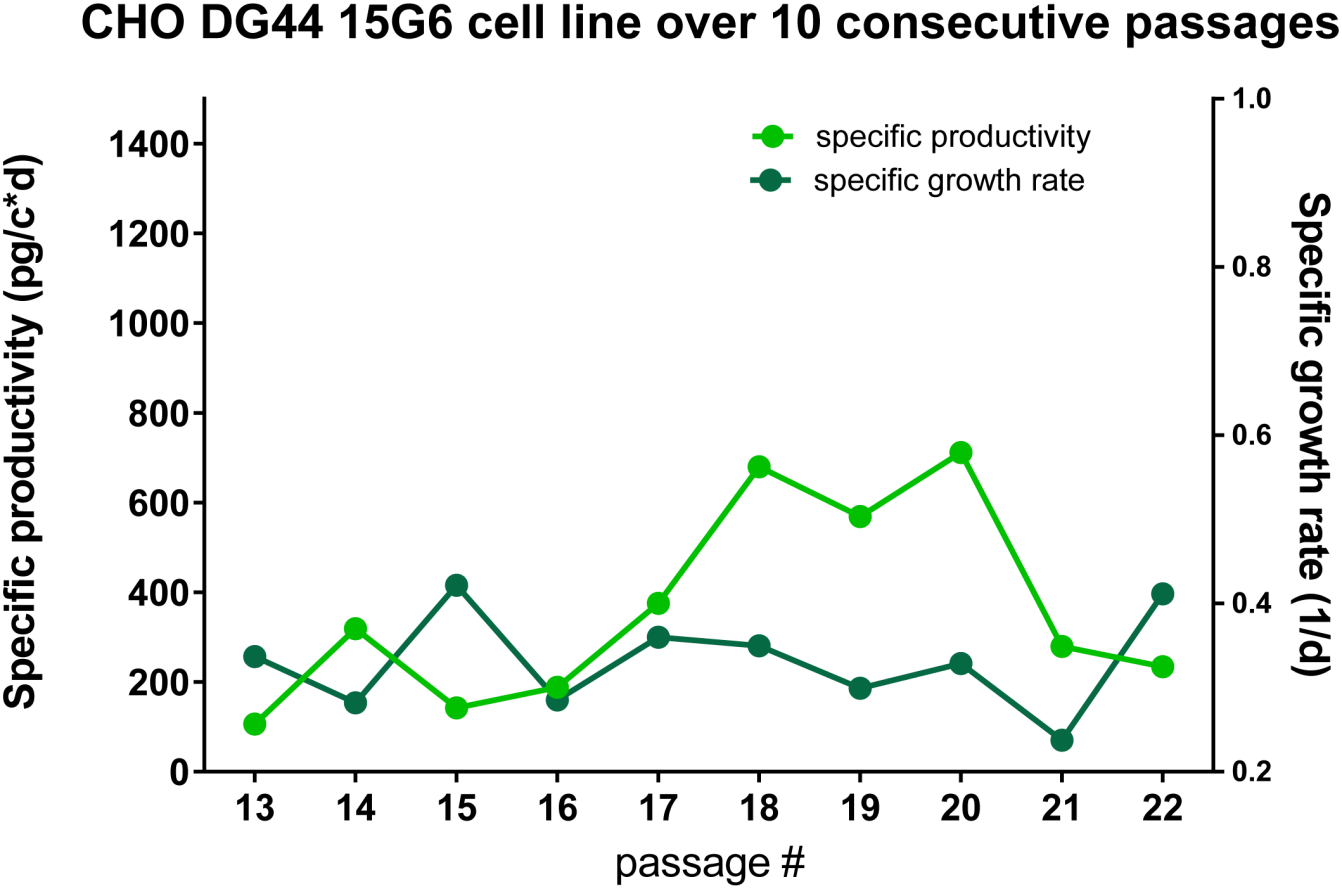
Display of specific productivity and specific growth rate of CHO DG44 SF cell line 15G6 over the span of 10 consecutive passages. Specific productivity rate is depicted in light green and specific growth rate is depicted in dark green color.

Plasmids containing the heavy and light chain sequences of CR9114 were constructed in a similar way as described before [15] using a bicistronic pIRES vectors. The CHO DG44 SF cell line was co-transfected with the plasmid containing the mAb CR9114 HC and the plasmid containing the mAb CR9114 LC in a 1:1 (Fig 2, called from here on CR9114-like, CR9114L) ratio using Lipofectamine^®^ 2000 in a 6-well plate format. 48 hours post transfection the selection process started in 96-well plates and cells were seeded at 7x10^3^ cells/well. Screening by several subsequent rounds of ELISAs started 3 weeks later. After the first screening round the selected clones were expanded into 24-well plates and MTX amplification pressure was increased to 200 nM. With several more rounds of screening and expansion it was decided not to increase the amplification pressure anymore before the cells fully recover (Fig 2). For several passages after the expansion into T-flasks, the culture was forming clumps and growing slowly, making it difficult to measure the cell count and viability reliably. Therefore, we decided to cultivate the cells in shaking flasks at 37°C, 7.5% CO_2_ and 150 rpm and the selection medium was further supplemented by 1% Pluronic F68. Cell count, viability and ELISA titers were determined with each passage and an overview of specific growth rate and specific productivity over 10 consecutive passages is displayed in Fig 3. The specific growth rate of the CR9114L-producing cell line (clone 15G6) with an average of μ = 3.3 1/d is lower as compared to the parental cell line. The specific productivity averages at around 360 picogram per cell per day (pgc^-1^d^-1^), which resulted in approximately 25 mg/L antibody yield from the supernatant on average (Fig 3). In the last two monitored passages, the specific growth rate started to increase and the specific productivity went down, possibly implying that an additional subcloning round might be necessary.

### Purification and *in vitro* testing of stably expressed mAb CR9114L

**Fig 4:**
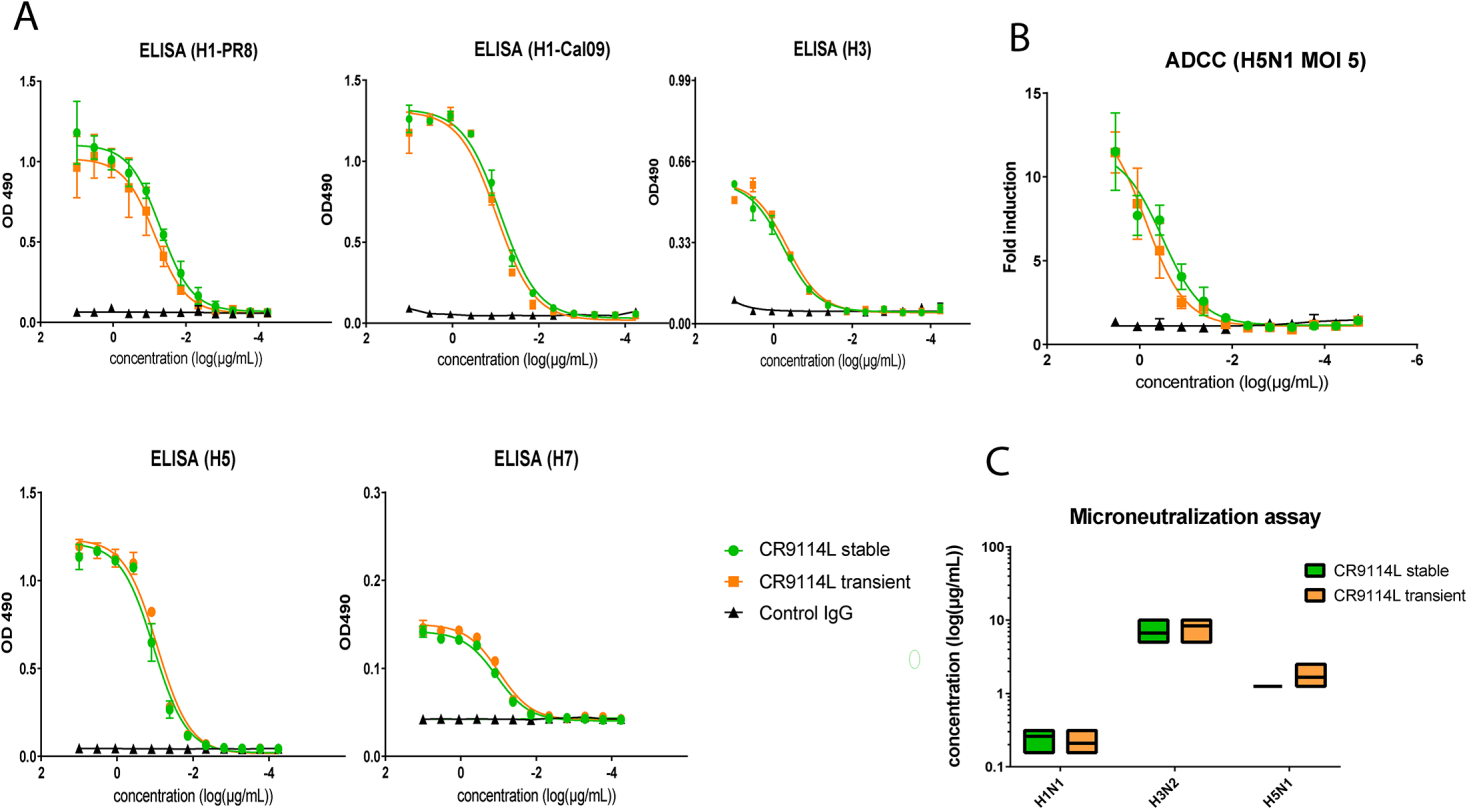
*In vitro* testing of purified CR9114L mAb expressed stably by cell line CHO DG44 SF 15G6 and transiently by Expi293F cells. A) ELISA assay using plates coated with 2 μg/mL of recombinantly expressed A/PR/8/34 HA (H1), Cal09 HA (H1), A/**I**ndiana/10**/**11 (H3), A/chicken/Netherlands/14015531/14 (H5) and A/chicken/Italy/13474/99 (H7). B) ADCC reporter assay demonstrates high level of similarity between stably and recombinantly produced CR9114L mAbs. The graph shows the fold induction over the negative control. Data pooled from two technical runs. C) Microneutralization assay results representing the minimal concentration of monoclonal antibody at which neutralization of the virus (A/Netherlands/602/2009 (H1N1), A/Philippines/2/1982 (H3N2) and A/Vietnam/1203/04 (H5N1)) occurs, thus preventing agglutination. Data pooled from two technical runs.

Supernatants from the individual passages were pooled, filtered through a 0.22 um filter and purified via a protein G column using an AKTA Pure L25 system. Purified CR9114L mAb from the 15G6 cell line was tested in ELISA, on plates coated with A/PR/8/34 HA (H1), Cal09 HA (H1), A/**I**ndiana/10**/**11 (H3), A/chicken/Netherlands/14015531/14 (H5) and A/chicken/Italy/13474/99 (H7) side by side with a CR9114L mAb purified from a transient transfection of Expi 293F cells. Both mAbs showed very similar binding profiles (Fig 4A) and IC_50_ values. Effector functions were assessed *in vitro* in ADCC reporter and microneutralization assays using an A/Vietnam/1203/04; PR8 6:2 (H5N1, low path), A/Netherlands/602/2009 (H1N1) or A/Philippines/2/1982 (H3N2) influenza virus. Stably expressed CR9114L mAb manifested similar activity profiles as transiently expressed CR9114L mAb, depicted as the fold induction over the negative control in ADCC (Fig 4B) and as minimal concentration needed for the neutralization to occur (Fig 4C).

## Discussion

MAb CR9114 is widely used and up to this point it is the only monoclonal antibody considered to be a universal binder of all influenza A and B virus HAs. Moreover, the recent change of focus to antibodies’ effector functions in the context of infectious diseases—and especially influenza virus [2, 4, 16–21]—is making mAb CR9114 the ideal positive control in any assay attempting to assess the ADCC, phagocytic or complement activating potential of newly discovered antibody candidates. Positive control mAbs generated through transient transfection show batch to batch variation and producing antibody through this method is tedious and expensive. Establishing a stable cell line offers a solution for these problems. Therefore we generated a stable, serum free CHO-DG44 based cell line producing a mAb CR9114L. While the clone expresses the published variable HC and LC regions of CR9114, its constant regions are from a generic human IgG1. Therefore, the mAb was termed CR9114-like or CR9114L.

To generate the cell line, we first adapted a CHO DG44 cell clone to a serum-free conditions using a step-wise approach. With the FBS concentration reaching 2% and below, cells were no longer able to recover in terms of viability and specific growth rate when cultivated in DMEM/Ham’s F12 medium despite the added supplements. At this point we started to search for the best suitable alternative amongst the commercially available media designed specifically for CHO cultures, such as ProCHO5, CD DG44, OptiCHO and CHO-S-SFM II. In our approach, CHO-S-SFM II medium provided the best conditions for newly established serum-free culture and cells ultimately regained viability above 90% and a stable specific growth rate. It is possible, if provided a longer time for adaptation to new conditions between individual FBS reduction steps, more than one of the tested media would be able to accommodate the needs of our CHO DG44 SF culture. This serum free cell line was then transfected with plasmids coding for CR9114L, a neomycin resistance marker and the DHFR gene which host CHO DG44 cells lack. Selection pressure and subcloning resulted in clone 15G6 which produces supernatants with antibody titers that have been consistently reaching ~25 mg/L over the span of several months, a production rate that rivals the production rate of many hybridoma cell lines. However, the potential of this cell line is still not fully exploited since MTX concentrations are currently just at 200 nM. With additional rounds of MTX amplification and subcloning, the production rate could be further increased using MTX concentrations up to 500 nM. It has been observed before that higher concentrations of MTX not necessarily result in higher specific productivities [22].

Stably expressed CR9114L mAb was compared side by side with a transiently expressed CR9114L in ELISA, ADCC and microneutralization assays. There are small deviations between the antibodies which might stem from a different glycosylation pattern between CHO DG44 cells and 293F cells, despite the CHO glycosylation pattern being very similar to human glycosylation patterns. Overall, binding profiles in ELISA, neutralizing potency in a microneutralization assay and the ability to mediate ADCC are very similar between these two monoclonal antibodies. Moreover, during the process of selection and expansion, CR9114L mAb demonstrated binding against a range of different recombinantly expressed HAs, further confirming the similarity to the originally described CR9114 mAb [1].

In summary, we adapted CHO DG44 cell line to serum free conditions, transfected it with CR9114L expressing plasmids and selected a healthy, stable clone, 15G6, that shows robust antibody production. Antibody produced by this clone showed activity in binding and functional assays comparable to mAb produced by transient expression in 293F cells. The established cell line will be a useful tool for academic influenza virus research.
